# Observation of treated iris neovascularization by swept-source-based en-face anterior-segment optical coherence tomography angiography

**DOI:** 10.1038/s41598-019-46514-z

**Published:** 2019-07-16

**Authors:** Daiki Shiozaki, Susumu Sakimoto, Akihiko Shiraki, Taku Wakabayashi, Yoko Fukushima, Yoshinori Oie, Shinichi Usui, Shigeru Sato, Hirokazu Sakaguchi, Kohji Nishida

**Affiliations:** Department of Ophthalmology, Osaka University Graduate School of Medicine, 2-2 Yamadaoka, Suita, Japan

**Keywords:** Optical imaging, Neuroscience

## Abstract

We evaluated regression of iris neovascularization (INV) using en-face anterior-segment optical coherence tomography angiography (AS-OCTA) after anti-vascular endothelial growth factor (VEGF) therapy. Seven consecutive eyes with INV were examined before and after anti-VEGF therapy, and all AS-OCTA scans were obtained using a swept-source OCTA system with an anterior-segment lens adapter. Slit-lamp microscopy photography and anterior indocyanine green angiography also were performed. Quantitative analyses of the vascular density, vascular lacunarity, and fractal dimension on AS-OCTA images were performed. AS-OCTA visualized the INV as signals around the pupillary margin, which corresponded to the vasculature confirmed by slit-lamp microscopy. After anti-VEGF drug injection, regression of INV was observed by AS-OCTA in all eyes (100%). The vascular density decreased and vascular lacunarity increased significantly after anti-VEGF therapy. This pilot study demonstrated the ability of AS-OCTA not only to detect but also to evaluate INV. Further study is warranted to improve the algorithm for delineating the iris vasculature to decrease artifacts.

## Introduction

Iris neovascularization (INV) and subsequent development of neovascular glaucoma are serious complications for patients with proliferative diabetic retinopathy (PDR), central retinal vein occlusion, and more^1^. Administration of anti-vascular endothelial growth factor (VEGF) agents or application of panretinal photocoagulation (PRP) are the first-line treatments for rubeosis irides^1,2^. We and other investigators have reported that anti-VEGF therapies such as intravitreal injections of bevacizumab (Avastin, Genentech Inc., South San Francisco, CA, USA), ranibizumab (Lucentis, Genentech, Inc.), and aflibercept (Eylea, Regeneron Pharmaceuticals, Tarrytown, NY, USA) have caused reduction or complete resolution of INV even after only a few days^2–4^. However, those studies were conducted using only slit-lamp microscopy and angiography. Use of slit-lamp biomicroscopy is unsuitable to quantify the INV. In addition, the diffuse dye leakage from the INV also hinders the exact measurement of vessel density.

Optical coherence tomography angiography (OCTA) is a noninvasive imaging modality that provides angiographic images for detecting motion contrast, which is created by reflectivity changes between multiple OCT B-scans of blood cell flow^5–7^. Compared to fluorescein angiography (FA), OCTA does not involve dye leakage and its images can be quantified. OCTA was used initially to evaluate posterior-segment diseases such as retinopathies or choroidal neovascularization^7,8^. More recently, when combined with a specialized attachment device, non-contact OCTA was introduced to visualize corneal and anterior-segment vessels^9–12^. Roberts *et al*. observed INV in their spectral-domain (SD)-based anterior-segment OCTA (AS-OCTA) images in a pilot study^12^. Moreover, a swept-source-based OCTA (SS-OCTA) machine visualized vessels even in the anterior segment, i.e., the cornea, conjunctiva, sclera, and iris^11^. Compared to SD-OCTA, SS-OCTA does not need a spectrometer, which introduces signal loss in the deeper windows^13^. Thus, SS-based AS-OCTA can visualize the vasculature through the opacities.

Corneal and conjunctival vessels are well detected using conventional slit-lamp microscopy. However, currently no non-invasive modality can quantify vessel distributions in the iris, and few reports have been published on the efficacy of SS-based AS-OCTA for detecting INV or iris vessels. In the current study, we used anti-VEGF therapies to treat eyes with INV to validate use of SS-based AS-OCTA in the iris and compared the OCTA signals from the INV before and after treatment.

## Results

Neovascular glaucoma, defined as intraocular pressues (IOPs) ranging from 24 to 59 mmHg, was unresponsive to antiglaucoma medications and was complicated in five (71.4%) eyes. All study eyes had been treated with PRP, and no eye had undergone a previous vitreous surgery. The patient characteristics and primary outcomes are shown in Table 1. The mean number of previous anti-VEGF injections was 2.0 ± 1.6 (range, 0–5). Six eyes had been treated with aflibercept (85.7%) and one eye with bevacizumab (14.3%).

**Table 1:** Patient demographic data. logMAR = logarithm of the minimum angle of resolution; VA = visual acuity.

Number of patients	6
Number of eyes	7
Age	66.0 ± 8.6
**Gender**	
Male (%)	4 (66.6)
Female (%)	2 (33.3)
**Causative disease (%)**	
Proliferative diabetic retinopathy	5 (71.4%)
Central retinal vein occlusion	1 (14.3%)
Central retinal artery occlusion	1 (14.3%)
Number of previous anti-VEGF agent injections	2.0 ± 1.6
**Number of patients who treated with an anti-VEGF agent (%)**	
Bevacizumab	6 (85.7%)
Aflibercept	1 (14.3%)
Pre-injection VA (logMAR)	1.07 ± 1.02
Pre-injection IOP (mmHg)	33.4 ± 17.1
Post-injection VA (logMAR)	1.12 ± 1.10
Post-injection IOP (mmHg)	15.9 ± 5.8
Days of image acquisition after last anti-VEGF drug injection	20.1 ± 11.9

**Figure 1:**
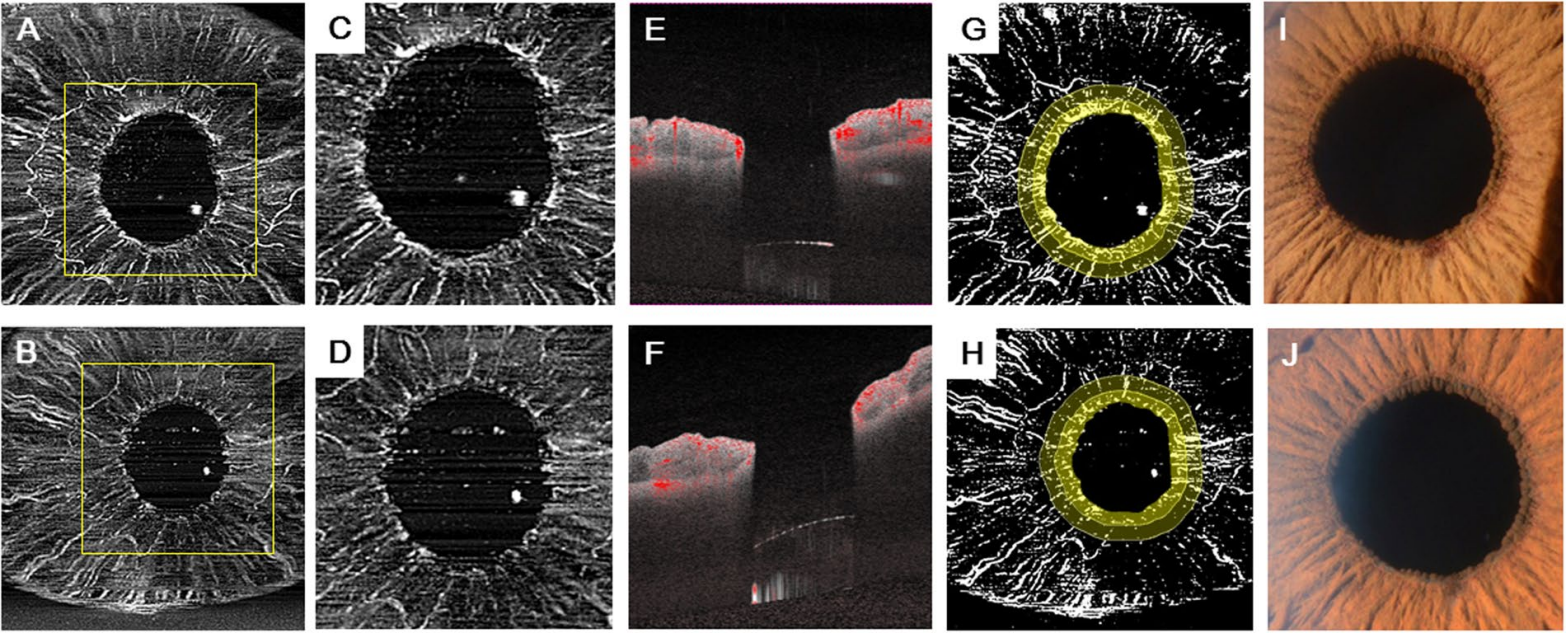
AS-OCTA performed to visualize INV treated with VEGF therapy. A 76-year-old man has PDR associated with INV in the right eye. (**A,C**) The AS-OCTA images were obtained before anti-VEGF drug injection. (**B,D**) The en-face AS-OCTA images show that the signals from the INV decreased 7 days after the injection. (**C,D**) High-magnification images corresponding to the yellow squares in the (**A**,**B**) respectively. (**E,F**) The flow dots of the B-scan images before (**E**) and after (**F**) anti-VEGF drug injection. (**G,H**) Binarized images obtained by (**A**,**B**) respectively. Diameters of 0.3 mm (dark yellow) and 0.6 mm (both dark and light yellow) diameter ring-shaped area were calculated. (**I, J**) Magnified slit-lamp microscopy images before (**I**) and after (**J**) anti-VEGF drug injection.

**Figure 2:**
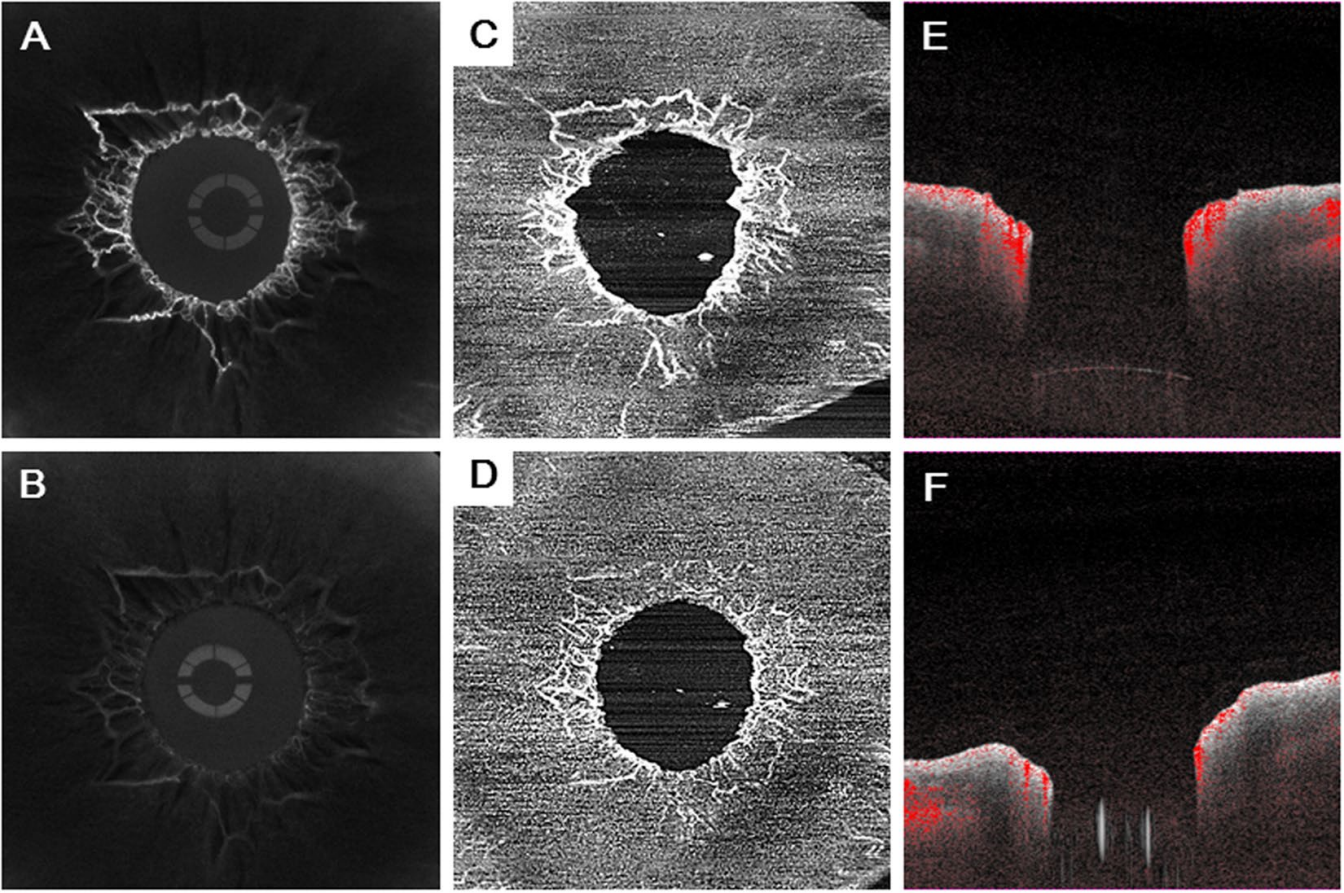
Comparison of anterior ICGA and AS-OCTA performed to visualize INV treated with anti-VEGF therapy. A 79-year-old man has PDR associated with INV in the right eye. (**A**) An ICGA image and (**C**) an en-face AS-OCTA image show the INV clearly around the pupil before anti-VEGF therapy. (**E**) Flow dots are seen in a B-scan OCTA image before therapy. (**B**) An ICGA image and (**D**) an en-face AS-OCTA image 7 days after anti-VEGF therapy demonstrate regression of INV. (**F**) Flow dots are seen in a B-scan OCTA image after therapy.

First, we confirmed the presence of iris rubeosis that was detected by slit-lamp microscopy or indocyanine green angiography (ICGA) and corresponded to that seen on the AS-OCTA images (Figs 1, 2). INV was detected by AS-OCTA as signals running around the pupillary margin, which corresponded to the vasculature confirmed by slit-lamp microscopy. The OCTA images were obtained successfully in all seven (100%) eyes before and after anti-VEGF therapy to treat INV. The INV appeared to be sprouting from the pupillary margin and was distinguishable from the normal iris vasculature that ran linearly and radially (Figs 1, 2). After intravitreal anti-VEGF drug injections, regression of INV was observed by AS-OCTA in all (100%) eyes (Figs 1, 2).

**Figure 3:**
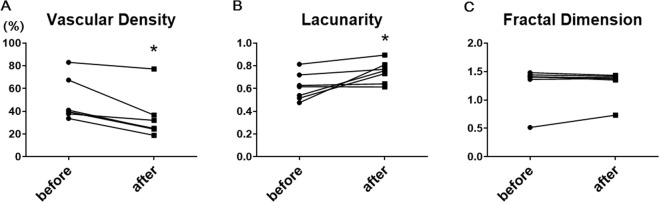
Slope charts for quantitative analysis for iris vascular parameters in a 0.3-mm diameter ring-shaped area imaged by AS-OCTA after anti-VEGF therapy. (**A**) Vascular density analysis shows a significant decrease in vascular density in the INV after anti-VEGF therapy (*P* = 0.025). (**B**) Analysis of vascular lacunarity shows a significant increase after anti-VEGF (*P* = 0.035), but the fractal dimension (**C**) does not show any change.

The vascular density, vascular lacunarity, and fractal dimension data are shown in Table 2. In the 0.3-mm-diameter ring area, the vascular density and vascular lacunarity changed significantly after anti-VEGF therapy, but no significant changes were seen in the 0.6-mm-diameter ring area. The average vascular density in the 0.3-mm-diameter ring area was 48.9 ± 18.7 before treatment and significantly decreased to 34.1 ± 19.9 (*P* = 0.025) after treatment. The average vascular lacunarity in the 0.3-mm-diameter ring area was 0.62 ± 0.12 before treatment and significantly increased to 0.76 ± 0.01 (*P* = 0.035) after treatment. However, the average fractal dimension in the 0.3-mm-diameter ring area was 1.29 ± 0.34 before treatment and 1.30 ± 0.25 after treatment, which did not reach significance (*P* = 0.406) (Fig. 3, Table 2).

**Table 2:** Quantitative analysis of iris vascular parameters imaged by AS-OCTA after anti-VEGF therapy.

	0.3-mm-diameter ring area			0.6-mm-diameter ring area		
	Pre	Post	*P* value	Pre	Post	*P* value
Vascular density (%)	48.9 ± 18.7	34.1 ± 19.9	0.025	39.6 ± 19.3	29.6 ± 19.8	0.085
Vascular lacunarity	0.62 ± 0.12	0.76 ± 0.01	0.035	0.72 ± 0.14	0.83 ± 0.17	0.248
Fractal dimension	1.29 ± 0.34	1.30 ± 0.25	0.406	1.53 ± 0.02	1.51 ± 0.03	0.253

Pre = pre-injection; post = post-injection.

## Discussion

The current study evaluated the efficacy of SS-based AS-OCTA for detecting INV in eyes with ischemic proliferative retinopathy. After anti-VEGF drug injections, the vascular density and vascular lacunarity, which were quantified with AS-OCTA, decreased significantly in eyes with INV. Thus, visualization of INV by non-invasive OCTA combined with an attachment module for the anterior segment of the eye was efficacious in this study.

We first investigated the regression of INV with anti-VEGF therapy that reduces INV significantly^2,3^, which enabled the AS-OCTA assessment to detect the vasculature changes. After the anti-VEGF drug injections, the vascular density decreased and vascular lacunarity increased significantly in eyes with INV. Another interesting finding was that the fractal dimension did not change even though the actual en-face OCTA images seemed to show that the vessels regressed. The reasons for this might have been the configuration of the INV and the multiple types of artifacts. INV regression is too subtle to be reflected in the change of the fractal dimension. Regarding the artifacts, multiple artifacts, such as motion and projection artifacts, in OCTA performed to visualize the retinal vasculature have been reported; however, clarifying the mechanism and integration of knowledge about the artifacts in AS-OCTA images is beyond the scope of this study and should be investigated in future studies.

Several groups have reported the clinical relevance of imaging the anterior segment of the eye using AS-OCTA, which is based on SD technologies^9,12,14,15^. A novel finding in the current study concerned the adoption of SS-based OCTA to analyze the INV, because in contrast to SD-OCTA, the SS technology used in the current study used a tunable, narrow-band laser, by which the wavelength can be converted electronically to high speed^16^. This means both that no spectrometer was required, which would have introduced significant signal loss with window depth that is being used in SD-OCT, and that the SS-OCTA provides a better image regarding the signal-to-noise ratio, especially in the deeper tissues. Another reason is that, while SD-OCT has speeds that typically are 70 to 80 kHz and no greater improvement in speed is expected, SS-OCTs run at 100 kHz, which provides significantly faster acquisition times, and is less sensitive to eye movement or iris contraction^17^. Finally, SS-OCT uses a longer wavelength, i.e., 1,050 nm, which also provides optical penetration even through corneal edema or into pigmented iris^18^.

To detect INV, the current modality involves slit-lamp biomicroscopy, gonioscopy, and FA or ICGA^12,19–21^. Use of slit-lamp biomicroscopy or gonioscopy is unsuitable to quantify the INV. Dye-based technologies, such as FA or ICGA, are effective for detecting INV; however, complications associated with administration of fluorescein can develop^20^. In addition, the diffuse dye leakage from INV also hinders the exact measurement of vessel density. One of the advantages of OCTA is clear visualization of vessels not obscured by dye leakage. Moreover, dye-based iris angiographies usually are performed under mitotic conditions; however, the original ischemic retinopathy that is the cause of INV should be examined through dilated pupils during the same visit^19^.

In the current study, all subjects were Asian and had dark irises. Based on a preliminary study by Roberts *et al*.^12^, differentiating INV using OCTA might be easier in eyes with darker irises than in eyes with light irises. The vessel patterns of the iris acquired by OCTA were similar to those in the ICGA images reported previously^3^.

One of the most important limitations of the current study was the image quality of the AS-OCTA machine. Even though SS-based OCTAs can obtain images faster, more motion artifacts were created than when obtaining retinal scans. As mentioned previously, multiple types of artifacts can affect image quality^22^. An appropriate algorithm specific to the anterior segment and multiple image averaging might improve the AS-OCTA images. In addition, segmentation of the vascular layer in the iris also is warranted because layer segmentation analysis is believed to be the most significant advantage of the OCTA, which was not accomplished in the current modality.

In conclusion, in the current pilot study, we demonstrated the ability of AS-OCTA to detect and possibly quantify the function of the INV. A study with more patients is needed to improve the algorithm for imaging the iris vasculature to decrease artifacts.

## Methods

The Institutional Review Board of Osaka University Hospital approved this retrospective, institutional, cross-sectional pilot study, which adhered to the tenets of the Declaration of Helsinki. Informed consent was obtained from all participants.

Seven eyes of six Japanese patients (4 men, 2 women; mean age, 66.0 ± 8.6 years) with INV associated with PDR (5 eyes), central retinal vein occlusion (1 eye), and central retinal artery occlusion (1 eye) who visited the Osaka University Hospital, Osaka, Japan, were enrolled in this study.

### Examinations

All subjects underwent imaging using the SS-OCTA system (PLEX Elite 9000, Carl Zeiss Meditec, Dublin, CA, USA), which was equipped with an anterior-segment optical adaptor with a 10-diopter optical adaptor lens (Carl Zeiss Meditec). This instrument has a central wavelength between 1,040 and 1,060 nm, a bandwidth of 100 nm, an A-scan depth of 3.0 mm in tissue, and a full width at half-maximal axial resolution of about 5 mm in tissue. The instrument captures 100,000 A-scans/second^11^. We also measured the best-corrected visual acuity (VA) and IOP. High magnification slit-lamp microscopy photography also was performed in all subjects.

### AS-OCTA image acquisition and processing

For each subject, a 3 × 3-mm scan pattern was used to acquire AS-OCTA images of the iris, which consisted of 300 A-scans/B-scan repeated four times at each of the 300 B-scan positions. The size of this 3 × 3-mm scan pattern corresponds to typical retinal dimensions and was approximately 6 × 6 mm in the AS-OCTA images with a digital lateral sampling of about 20 microns/pixel (personal communication from Gerd Klose from Carl Zeiss Meditec). En-face images were generated using built-in software (version 1.6.0.21130, Carl Zeiss Meditec). Flattening was performed at the level of the conjunctival epithelium, which was misidentified as the inner limiting membrane by the software. The projection-resolved algorithm in the built-in software was used when developing the deep-layer en-face image^11^.

### Quantitative measurements

Vascular density, vascular lacunarity, and fractal dimension were measured in the 3 × 3-mm scan images. For measurements, an inner pupil area was cropped using the polygonal tool from a 1,024 × 1,024-pixel original image obtained using ImageJ software (Wayne Rasband, National Institutes of Health [NIH], Bethesda, MD, USA). The software automatically cropped ring-shaped areas from the pupillary margin that were 50 or 100 pixels in diameter and corresponded to about 0.3 or 0.6 mm on the actual scan. The images of those cropped ring-shaped areas then were binarized using a modified version of the previously reported procedure^23,24^ (Fig. 1G,H). The images were converted to 8 bits and assigned a value of 255 (complete white) to all pixels with a positive gray level and a value of 0 (complete black) was assigned to the others using the contrast auto local threshold method (radius, 75 pixels; parameter 1, default; parameter 2, default)^25^. The vascular density was defined as the ratio of the area occupied by the vessels divided by the total area. Vascular lacunarity and fractal dimension were calculated using the ImageJ plugin FracLac software (Wayne Rasband, NIH). Vascular lacunarity, which characterizes oddities identified when vessel organization is disrupted significantly, may be useful to characterize and quantitatively analyze vascular networks in drug-treated specimens^26^. Vascular lacunarity and fractal dimension were calculated on the binarized image (Fig. 1G,H) using the ImageJ plug-in software (FracLac). The box-counting method was used for the calculation. The vascular lacunarity and fractal dimension could range from 0 to 2, and images with a more complex vessel branching pattern would have a higher fractal dimension.

### Anti-VEGF therapy

The patients underwent intravitreal injections of aflibercept (2 mg) or bevacizumab (1.25 mg). Examinations before administration of therapy were performed just before the injection at the same visit, and examinations after therapy were performed longer than 1 week and shorter than 4 weeks after therapy; patients were followed for more than 2 months.

### ICGA

One patient, a 79-year-old man, underwent ICGA using a fundus camera (TRC-50DX, Topcon Corporation, Tokyo, Japan) as described previously^27^. Briefly, pharmaceutical-grade ICG (Ophthagreen® 25 mg, Santen, Tokyo, Japan) was dissolved in 2.0 mL of the manufacturer-supplied distilled water. After the ICG was injected, fluorescent images were acquired with the anterior-segment module.

### Statistical analysis

For VAs worse than 20/400, the logarithm of the minimum angle of resolution values of 2.0, 2.3, 2.6, and 2.9 were assigned for counting fingers, hand motions, light perception (LP), and no LP, respectively^14^. The data were analyzed using GraphPad Prism (GraphPad Software, La Jolla, CA, USA). The paired t-test or Mann–Whitney U test was performed as appropriate. *P* < 0.05 was considered significant.
